# Assessment of physical properties of bioactive glass-modified universal multimode adhesive and its bonding potential to artificially induced caries affected dentin

**DOI:** 10.1186/s12903-024-04175-z

**Published:** 2024-04-05

**Authors:** Nada E. Kazem, Dina A. El-Refai, Ghada Alian

**Affiliations:** Dental Biomaterials Department, Faculty of Dentistry, Ain Shams University, African Union Organization Street, Abbasia, Cairo 11566 Egypt

**Keywords:** Bioactive glass, Micro-tensile bond strength, Degree of conversion, pH of adhesive, Viscosity, Bioactivity

## Abstract

**Background:**

To evaluate the physical properties of bioactive glass-modified universal multimode adhesive and its micro-tensile bond strength (µTBS) to artificially induced caries-affected dentin.

**Methods:**

All bond universal adhesive was used in the study. Specimens were divided into 2 main groups: control unmodified adhesive and 5 wt% BAG modified adhesive. The degree of conversion, pH, bioactivity, and viscosity of the adhesives were tested with *n* = 5 for each test. Micro-tensile bond strength evaluation was done in etch & rinse (ER) and selective-etch (SE) modes, where 24 human molar teeth were used (*n* = 3), 12 teeth for immediate bond strength, and the other 12 were tested after 6 months of storage in simulated body fluid (SBF).

**Results:**

No significant difference was found between the control and the 5wt% BAG groups regarding the degree of conversion (61.01 ± 0.43 and 60.44 ± 0.61 respectively) and the viscosity (109.77 ± 22.3 and 124.3 ± 9.92 respectively). The control group revealed significantly lower pH values than the 5wt% BAG group (3.16 ± 0.5 and 4.26 ± 0.09 respectively). Immediate bond strength results revealed that the 5wt% BAG in the ER mode had the highest bond strength followed by the control group in the ER mode (44.16 ± 7.53 and 44.00 ± 7.96 respectively). SE groups showed that the immediate strength of the 5wt% BAG group was higher than the control group (42.09 ± 6.02 and 39.29 ± 6.64 respectively). After 6 months of storage, bond strength results revealed a decrease in bond strength values for the control groups but not for the 5wt% BAG in both application modes.

**Conclusions:**

The incorporation of BAG (5wt%) improved the universal adhesive micro-tensile bond strength and bond durability for both adhesive application modes without affecting its degree of conversion or viscosity.

## Background

The most common pathological change affecting dentin is dental caries. Carious dentin is composed of two main layers. The first (outer) layer is bacterially infected, highly demineralized, difficult to remineralize, and consists of collagen fibrils that are irreversibly denatured due to the destructed cross-linkages. Meanwhile, the second (inner) layer is uninfected and known as caries-affected dentin (CAD). This layer is not entirely demineralized and can be remineralized and should be preserved during caries treatment [1]. Therefore, during cavity preparation, the caries-infected dentin is removed, and the cavity floor is mostly composed of CAD. Consequently, when using adhesive restorations the substrate is usually CAD, not normal dentin [1].

In 1969, Professor Larry Hench was the first to introduce the Bioactive glass (BAG), a silicate glass with sodium calcium phosphor. It presented a revolution in tissue engineering as it exhibited an excellent bonding ability to bone [2]. His discovery shifted the criteria of materials used for the replacement of tissues from bioinert to bioactive. A bioactive material could interact favorably with host tissues driving its regeneration and repair to become of utmost need. From this perspective, bioactive materials comprising calcium phosphate, hydroxyapatite, bioactive glass, and calcium silicates have increased [3].

After the invention of BAG, it was broadly researched to reach the reason behind its high bioactivity [4]. The mechanism for its bone-bonding ability was due to the leaching out of ions from the glass, forming carbonated calcium-deficient hydroxyl apatite (HCA) to bond to the tissues’ collagen [5]. Ever since, BAG has been extensively used in many procedures such as cartilage and bone repair and regeneration as well as implant coatings [2]. In dentistry, the leachable ions from BAG and the subsequent HCA formation can be used to remineralize the hard dental tissues. In-vitro and in-vivo findings confirmed that remineralization may be accomplished by raising the amount of mineral content, hardness, and modulus of elasticity of dental hard tissues [6, 7]. Moreover, Ryou et al. [8] revealed that the remineralization of demineralized zones in the hybrid layer may be a promising way for sustaining the endurance of the resin-dentin interface, as well as fossilizing the endogenous matrix metalloproteinases (MMPs) and the consequent protection of collagen fibrils [9].

Previously, BAG was directly applied to demineralized dental tissues for its remineralization [10]. Recently, trials have been made to achieve resin-based restorative materials that contain bioactive nanofillers.

In a recent study done by Rao et al., a commercial universal adhesive modified with poly(amidoamine) dendrimer (PAMAM) loaded mesoporous bioactive glass nanoparticles (A-PMBG) was used to assess the remineralization effect and microtensile bond strength of the modified adhesive to artificially induced CAD, and concluded that the microtensile bond strength increased after storage for 6 months [11]. This was attributed to the ability of bioactive glass particles to remineralize CAD along with its ability to act as an excellent delivery system to PAMAM polymer which could bind to collagen fibrils, enhancing the remineralization process [11].

Another study assessed the effect of adding 10 and 20 wt% bioglass powder to a nanofilled universal adhesive, and the results showed that the micro-tensile bond strength results of both concentrations were inferior to the control group [12]. This might be attributed to the high amount of bioglass powder used in an already-filled universal adhesive, which in turn might have affected the viscosity and flow properties of the adhesive and its ability to infiltrate dentin.

Thus, this study was conducted to introduce the use of bioactive nanofillers in universal adhesives, to gain the benefit of their remineralizing effect, especially on mineral-depleted areas at the resin-dentin interface.

The null hypothesis tested was that adding BAG would not affect the degree of conversion, pH, and viscosity of the universal adhesive as well as its bonding potential and bond durability to demineralized dentin in either ER or SE mode.

## Methods

### Materials

Materials used in this study are mentioned in Table 1.

**Table 1:** Materials used in the study, their brand names, descriptions, manufacturers, and lot numbers

Material	Description	Composition	Manufacturer	Lot number
All bond universal	Ultra Mild Universal multi-mode adhesive system pH (3.2)	10-MDP, Bis-GMA, HEMA, Ethanol, Water, Initiators	Bisco; Schaumburg, IL, USA	2,100,006,177
Grandio	Universal nanohybrid resin composite	Bis-GMA, UDMA & TEG-DMA matrix Filler content: 87 wt% / 71.4 vol% inorganic filler loading	Voco, Cuxhaven, Germany	2,050,486
B&E etch	Etchant delivery system	37%Phosphoric Acid Semi Gel	B&E, Korea	BEE220003
Bioglass nanoparticles	10 nm powder particles	45% Silica, 25% CaO, 25% Na_2_O & 5% P_2_O_5_	Nanostreams, UK	NS0001

### Methods

**Fig. 1:**
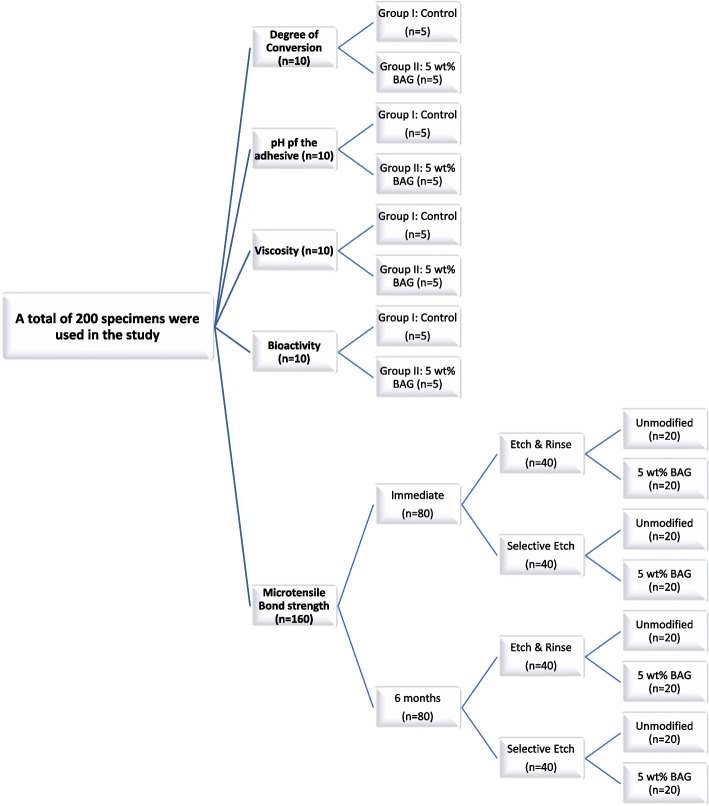
Flowchart for the test groups

A flowchart showing specimen grouping is presented in Fig. 1.

### Preparation of the experimental adhesive

Bioactive glass (BAG) nanoparticles were added to Universal adhesive (All-Bond Universal, Bisco, Schaumburg, USA) to obtain 5% (w/w) based on a pilot study. 1 ml of adhesive was freshly prepared for each test. The adhesive was first weighed using 4 digits, digital analytical balance (AZ 214, Sartorius AG, Germany), and then BAG powder was added to give a concentration of 95% (w/w) Adhesive: 5% (w/w) BAG powder.

The mixture was then placed in a light-proof bottle kept in a sealed bag and placed in an ultrasonic bath (MCS, Codyson, China) for 20 min to ensure homogenous distribution of the BAG nanoparticles [13].

### Degree of conversion measurement (DC)

A potassium bromide (KBr) disc was prepared for each specimen. Each disc was placed in the Fourier Transform Infrared (FTIR) spectroscopy (Nicolet 6700, Thermo Fisher, MA, USA) to obtain an absorbance spectrum of molecules present in the air. This was used as a background. For group I (unmodified Adhesive), the adhesive was applied on the KBr discs using a disposable micro-brush (*n* = 5). For group II (5 wt% BAG modified adhesive), a micro-brush tip was dipped in the BAG/adhesive complex. Then the complex was applied to the KBr discs (*n* = 5).

The absorbance spectrum of the unpolymerized adhesive in the two groups was recorded with the FTIR spectrometer. Then, these spectra were divided by the background spectrum. This step will remove all absorptions contained in the background and obtain a spectrum that belonged to the sample solely. The mid-infrared (MIR) region was used [14].

Following that, discs were covered with celluloid matrix, and another absorbance spectrum was obtained for the two groups after the adhesive was photo-cured for 10s using a LED light curing unit (Elipar S10, 3 M ESPE, Germany) with an output intensity of 1200 mW/cm^2^. The light cure unit tip was held 3 mm away from the disc guided by the thickness of the upper part of the holder.

The DC% was determined by measuring the ratio of absorbance intensities for aliphatic C = C and the internal reference of aromatic (C…C) at peaks 1638 cm^−1^ and 1608 cm-1, respectively. before and following the curing of the specimens^‎^, using the following equation [15]:

$$\begin{array}{c}\mathbf{DC}\boldsymbol\%\boldsymbol=\boldsymbol\;\mathbf1\boldsymbol\;\boldsymbol-\boldsymbol\;\left(\left[\mathbf C\boldsymbol\;\mathbf{aliphatic}\boldsymbol/\mathbf C\boldsymbol\;\mathbf{aromatic}\right]\boldsymbol\;\boldsymbol/\boldsymbol\;\left[\mathbf U\boldsymbol\;\mathbf{aliphatic}\boldsymbol/\mathbf U\boldsymbol\;\mathbf{aromatic}\right]\right)\boldsymbol\;\boldsymbol\times\mathbf{100}\\\mathbf C\boldsymbol\;\boldsymbol=\boldsymbol\;\mathbf{cured}\boldsymbol\;\mathbf{adhesive}\boldsymbol,\boldsymbol\;\mathbf U\boldsymbol\;\boldsymbol=\boldsymbol\;\mathbf{uncured}\boldsymbol\;\mathbf{adhesive}\end{array}$$

### pH measurement

pH of the adhesives to be tested was recorded with a pH meter (Horiba LAQUAtwin Compact, OneTemp, Japan). First, distilled water was used to clean the pH meter, then calibration was done using its buffer solutions and excess water was blotted off. A micropipette was used to apply a drop of the adhesive to the pH meter. Five measurements were recorded for each group and the mean value for each group was determined [13].

### Viscosity measurement

The viscosity of the groups was measured using a rheometer (Brookfield Rheometer Programmable Model DV-III, Brookfield Engineering Laboratories, INC, USA). The device encompasses a revolving cone and a fixed plate (cone angle 5, cone diameter 40 mm, and gap 0.2 mm) with a specimen filling the gap between them. Half ml of each adhesive sample (*n* = 5) was positioned on the plate, and the cone was lowered down to measure the viscosities of the samples in centipoise (cP) [16].

### Bioactivity

Disk-shaped specimens (5 mm diameter and 1 mm thick) of each group (*n* = 5) were prepared in a split teflon mold. Adhesives were dripped onto a celluloid matrix until they filled the mold, covered with another celluloid matrix, and light-cured for 40 s using a LED curing unit Elipar S10 (3 M ESPE) with an output intentsity of 1200 mW/cm^2^ [17]. Specimens were observed by scanning electron microscope (SEM) (ESEM, Model Quanta 250 FEG, Field Emission Gun); at a magnification of 1000x, at 2 different time intervals; firstly after 24 h then after 28 days of storage in Simulated body fluid (SBF) prepared according to the formula shown in (Table 2), in an incubator at 37 °C.

**Table 2:** Order, amounts, weighing containers, purities, and formula weights of reagents for preparing 1000 ml of SBF

Order	Reagent	Amount	Container	Purity (%)	Formula Weight
1	NaCl	8.035 g	Weighing Paper	99.5	58.4430
2	NaHCO_3_	0.355 g	Weighing Paper	99.5	84.0068
3	KCl	0.225 g	Weighing Bottle	99.5	74.5515
4	K_2_HPO_4_.3H_2_O	0.231 g	Weighing Bottle	99.0	228.2220
5	MgCl_2_.6H_2_O	0.311 g	Weighing Bottle	98.0	203.3034
6	1.0 M-HCl	39 ml	Graduated Cylinder	-	-
7	CaCl_2_	0.292 g	Weighing Paper	95.0	110.9848
8	Na_2_SO_4_	0.072 g	Weighing Bottle	99.0	142.0428
9	Tris	6.118 g	Weighing Bottle	99.0	121.1356
10	1.0 M-HCl	0–5 ml	Syringe	-	-

### Micro-tensile bond strength (µTBS)

Testing was done according to the ISO standards no. 11405:2015 [18]. A total of 24 freshly extracted human third permanent molars for therapeutic and orthodontic reasons were used for this test. Teeth were cleaned from any hard and/or soft deposits using an ultrasonic scaler and kept in distilled water containing 0.5% thymol for not more than 1 month.

The sample size was calculated using G*Power software with a power of 80%. Based on a pilot study a total of 160 samples subdivided into 20 samples per subgroup was sufficient.

#### Teeth preparation

A flat dentin surface was obtained by removing the occlusal enamel with a low-speed diamond saw under water coolant (Isomet 4000, Buehler, Lake Bluff, IL, USA). To create a standardized smear layer, 600-grit SiC paper was used for 1 min under water coolant [19].

Each tooth was then covered with two layers of fast-setting acid-resistant nail varnish leaving only a rectangular flat dentin surface exposed.

Each tooth was then immersed separately in the demineralizing solution consisting of (50 mM acetate buffer solution at pH 4.5 holding 2.2 mM each of KH_2_PO_4_ and CaCl_2_ and 0.5 ppm fluoride in the form of NaF) [20] prepared at the faculty of pharmacy, Ain shams university, for 4 days at 25^0^C without changing the solution, to create a CAD layer. The amount of demineralizing solution in ml for each tooth was calculated according to the following equation: Area of rectangular window x 2 [21].

After that, remnants of nail varnish were removed from the occlusal surface using a scalpel, and the teeth were thoroughly cleaned under running distilled water to remove any residues from the demineralizing solution or nail varnish.

#### Bonding procedure

A metal matrix with a clamp no. 1.552 (Tor VM, Russia) was secured around the teeth, and its height was checked using a periodontal graduated probe to ensure the presence of a 4 mm height available for composite build-up.

For the etch and rinse (ER) group, a 37% phosphoric acid etchant was first placed on the enamel margins for 30 s and on the dentin surface for 15 s, after which it was thoroughly rinsed for 30 s. For the selective-etch (SE) group, the 37% phosphoric acid etchant was selectively placed on the outer enamel margin only for 30 s afterward it was rinsed for 30 s. The surface was then gently blot-dried from the excess water to leave the surface visibly moist.

Two coats of the universal adhesive were rubbed on the flat tooth surface along with the active application of each coat for 20 s followed by gentle air drying for 10 s. The adhesive layer was then light cured for 10 s using a LED curing unit Elipar S10 (3 M ESPE, Germany) with a light output of 1200 mW/cm^2^ [22].

The resin composite was then applied to the tooth surface in 2 mm increments each photocured for 20s. The last increment was covered with a glass slide before curing and curing was done through the glass slide. This was done to obtain a flat composite surface and to inhibit the oxygen-inhibited layer creation formation.

To ensure that the storage solution would reach the pulp of the tooth, and hence the dentinal tubules, the apices of the molars were cut off using a diamond saw and the flow of the SBF was checked by inserting a side vented needle from one canal and ensuring the solution came out from the other canals [23].

Each specimen was then kept separately in a tube enclosing simulated body fluid (SBF) whose composition followed Kokubu et al. [24]. The solutions were changed weekly and kept in an incubator at 37^o^C.

#### Micro-tensile bond strength testing

After the predetermined storage period (1 day and 6 months), the root of each tooth was mounted in a chemically cured acrylic resin block [25]. The teeth were then sectioned longitudinally in X- & Y- directions using a sharp diamond Isomet saw to obtain specimens 0.9 × 0.9 mm thick and 8 mm in height. The saw was driven at a blade speed of 2600 rpm and a feed rate of 8.5 mm/min. The thickness of each beam was confirmed using a digital caliper.

The bonded beams were collected, and the remaining dentin was measured, beams with less than 2 mm dentin in height were excluded. The beams were separately glued to a metal micro-tensile fixture using a fast-setting cyanoacrylate adhesive. The beams were glued to position the bonded interface perpendicular to the applied load.

A universal testing machine (Instron 3365, Norwood, MA, USA) was employed to test the micro-tensile bond strength. Tensile forces were applied at a crosshead speed of 1 mm/min starting from zero N/m^2^ until failure and the bond strength was calculated in (MPa) automatically using the BlueHill3 software according to the following equation [13]:

$$\begin{array}{c}\boldsymbol{\mu}\mathbf{TBS}\boldsymbol\;\boldsymbol=\boldsymbol\;\boldsymbol(\mathbf F\boldsymbol/\mathbf A\boldsymbol)\\\mathbf{Where}\boldsymbol,\boldsymbol\;\mathbf F\boldsymbol:\boldsymbol\;\mathbf{Force}\boldsymbol\;\mathbf{at}\boldsymbol\;\mathbf{failure}\boldsymbol,\boldsymbol\;\mathbf A\boldsymbol:\boldsymbol\;\mathbf{Area}\boldsymbol\;\mathbf{of}\boldsymbol\;\mathbf{the}\boldsymbol\;\mathbf{bonded}\boldsymbol\;\mathbf{interface}\boldsymbol\;\boldsymbol(\mathbf0\boldsymbol.\mathbf{81}\boldsymbol\;\mathbf{mm}^{\mathbf2}\boldsymbol)\end{array}$$

**Fig. 2:**
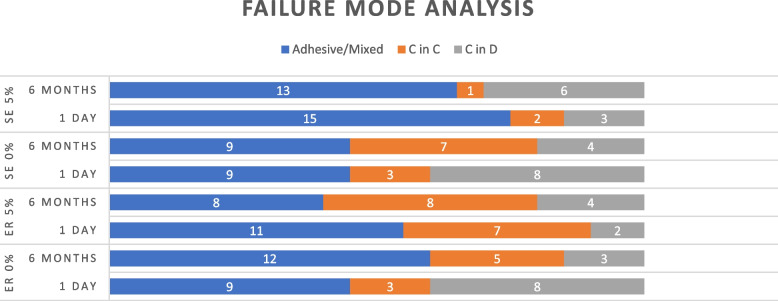
Failure mode analysis for different groups after microtensile bond strength testing

Failure pattern analysis was performed with a stereomicroscope at 40× magnification (SMZ 745T, Nikon, Japan), and fracture mode was classified as adhesive-mixed at the interface (A/M), cohesive in dentin (C in D); cohesive in composite resin (C in C) Fig. 2.

**Fig. 3:**
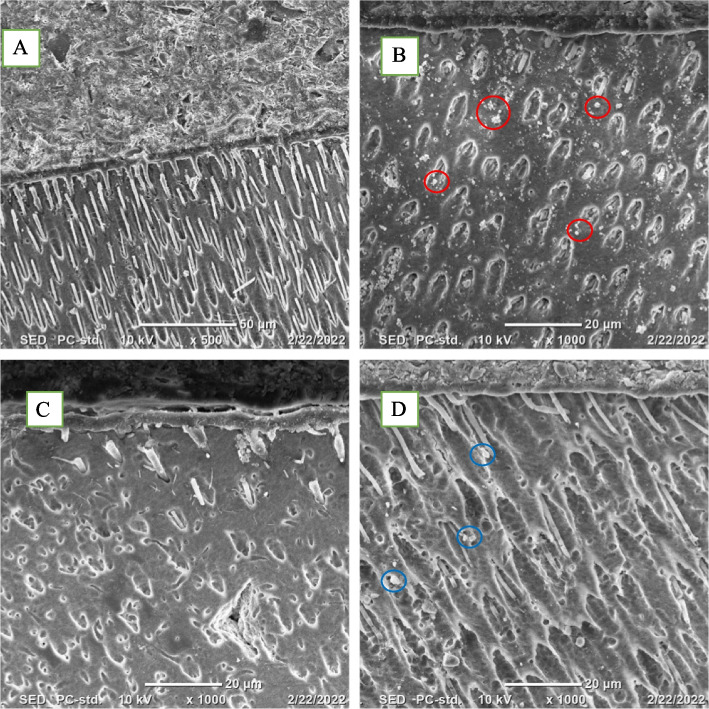
Representative SEM images for Hybrid layer assessment of **a** ER 0% 1 day, showing numerous and long resin tags, **b** ER 5% 1-day showing BAG particles penetration along with the adhesive (Red circles), **c** SE 0% 1 day showing few and short resin tags and **d** SE 5% 1 day showing BAG particles penetration inside the dentinal tubules (Blue circles)

Representative dentin-composite rod was taken from each of the 24-hour groups and scanned using SEM for hybrid layer assessment and to ensure penetration of the BAG particles along with the adhesive into the dentinal tubules (Fig. 3).

### Statistical analysis

Statistical analysis and data management were executed utilizing the Statistical Package for Social Sciences (SPSS) version 20. Numerical data were presented as mean and standard deviation values. Data were investigated for normality by verifying the data distribution and applying Kolmogorov-Smirnov and Shapiro-Wilk tests.

Comparisons among groups concerning normally distributed numeric variables were performed using an independent t-test. Repeated measures of ANOVA and paired t-tests were used to examine the impact of time. All *p*-values are two-sided. *P*-values ≤ 0.05 were considered significant.

## Results

### Degree of conversion results

No statistically significant difference was recorded between groups I (61.01 ± 0.43%) and II (60.44 ± 0.61%). The mean difference between both groups was (0.57 ± 0.33%) (Table 3).

**Table 3:** Mean ± standard deviation values of degree of conversion, pH, and viscosity for different groups

	Group I (Unmodified Adhesive)	Group II (5 wt% BAG modified adhesive)	*p*-value
Degree of conversion %	61.01 ± 0.43	60.44 ± 0.61	0.128 ns
pH	3.16 ± 0.05	4.26 ± 0.09	0.000*
Viscosity (cP)	124.30 ± 9.92	109.77 ± 22.30	0.236 ns

*ns *Non-significant

Significance level *p* ≤ 0.05, *significant

### pH results

Group II recorded a significantly higher value (4.26 ± 0.09) than that recorded in group I (3.16 ± 0.05), (*p* = 0.000). The mean difference between both groups was (1.1 ± 0.05) (Table 3).

### Viscosity results

No statistically significant difference was recorded between groups I (124.30 ± 9.92 cP) and II (109.77 ± 22.30 cP). The mean difference between both groups was (14.52 ± 10.91 cP) (Table 3).

### Bioactivity results

**Fig. 4:**
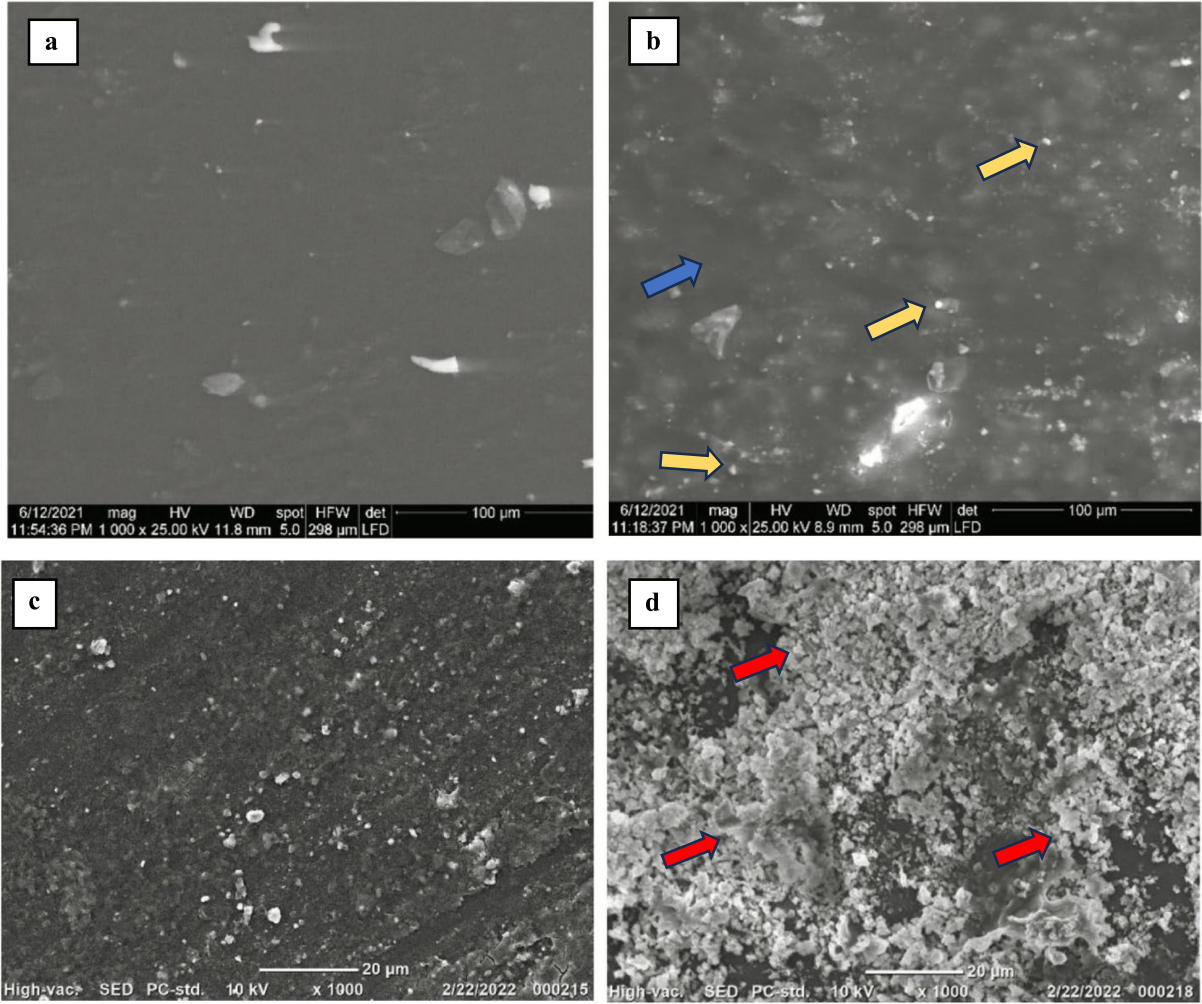
Representative SEM images showing **a **unmodified adhesive disc after 24 h, **b **5 wt% BAG modified adhesive disc after 24 h (Blue arrow: Organic matrix), (Yellow Arrow: BAG NPs), **c **unmodified adhesive disc after 28 days and d: 5 wt% BAG modified adhesive disc after 28 days (Red arrows: Crystals deposition)

SEM observation after 24 h revealed that group I showed a dark background which represents the organic matrix, whereas for group II there were opaque white nanoparticles representing the BAG nanoparticles dispersed in the organic matrix (Fig. 4).

After 28 days of immersion in SBF, specimens were gold sputtered and reobserved using SEM, scans showed the formation of crystal aggregates of various sizes and diameters and elevated above the surface, on the specimen surfaces of group II but not for group I (Fig. 4).

### Micro-tensile bond strength results

Mean and standard deviation values of micro-tensile bond strength values (MPa) are presented in Table 4.

In the control group, the µTBS mean values in ER and SE modes illustrated a statistically substantial decrease by time (*p* = 0.001) and (*p* = 0.002) respectively.

While in the 5 wt% BAG, no substantial difference was found in the ER and SE modes between different times (*p* = 0.608) and (*p* = 0.597) respectively.

**Table 4:** Mean ± standard deviation of µTBS values (MPa) and comparison between different observations within the same group (Repeated measures ANOVA test)

Adhesive.	Tech.	Immediate	6 months	*P*. value
		Mean ± SD	Mean ± SD	
Control	ER	44.00 ± 7.96 ^a^	31.98 ± 6.18 ^b^	0.001*
	SE	39.26 ± 6.64 ^a^	32.19 ± 8.18 ^b^	0.002*
5 wt% BAG	ER	44.16 ± 7.53 ^a^	42.82 ± 7.55 ^a^	0.608 ns
	SE	42.09 ± 6.02 ^a^	40.73 ± 9.49 ^a^	0.597ns

Post hoc Pairwise comparison: within the same row, means sharing the same letter is not significantly different

*ns* Non-significant

Significance level *p* ≤ 0.05, *significant

## Discussion

The durability and endurance of dental restorations are affected by the interaction between resin dental adhesives and dentin. Research executed on the adjustment of adhesive systems to decrease liability to degradation has suggested the addition of nanofillers to decrease hydrophilicity, reduce enzyme-aided degradation of collagen, and minimize stress contraction owing to reducing the polymeric matrix [26].

When a bioactive material interacts with the biological surroundings, it produces a favorable biological outcome. Among these outcomes is the establishment of an HA layer forming a bond between the tissue and the material. The discovery of BAG along with its bioactive properties caused a healthcare innovation where it’s used in multiple clinical trials concerning hard tissue regeneration. The intervention of nanotechnology helped the synthesis of BAG in the nanoscale [27].

The mechanism of action of BAG nanoparticles is based on an exchange process when placed in physiological fluids. Upon immersion, cation exchange of Na^+^ and Ca^2+^ by protons (H^+^ or H_3_O) occurs on the surface to form microporous silica (SiO_2_^−^-rich layer) upon which Ca and P ions are adsorbed and the HCA layer forms [28].

Preparation of the modified adhesive was based on ultrasonication of the experimental adhesive after incorporation of the BAG for 20 min. This was based on previous literature to obtain a homogenous distribution of the BAG NPs within the adhesive [13].

One of the most important factors for reliable and durable dental bonding is the DC of dental adhesives. A substandard DC will lead to a decreased interfacial strength and subsequent bonding instability and vulnerability [29].

It was evident that the presence of unreacted monomers inside the hybrid layer leads to the formation of a porous hybrid structure and increases the adhesive layer permeability [30]. High DC is correlated with an improvement in the mechanical properties of resin materials as well as higher biocompatibility [15, 31].

The addition of 5 wt% BAG did not influence the adhesive degree of conversion, and the DC remained almost the same for both groups. This finding concurred with other studies, where the addition of hydroxyapatite nanorods and niobium-based glass did not impact the adhesive degree of conversion [17, 29].

The pH or acidity of the bonding agent determines the extent of demineralization of the tooth structure. Therefore, the addition of another substance should not affect the pH of SE adhesives and preserve their etching ability [13].

In the current study, adding 5 wt% BAG significantly increased the pH of the adhesive; however, this did not affect the bond strength as the substrate used was demineralized dentin. This could be justified by the action of the demineralizing solution on dentin which caused partial etching of the surface, so the bond strength was not altered by the increased pH of the modified adhesive.

Dental adhesives must be in close contact with the substrate as well as having the ability to spread and flow easily on the surface to attain good adhesion. Adhesives with high viscosity will resist flow and will not perfectly wet the surface. On the other hand, adhesives which are too flowy are difficult to control during application on the substrate. Therefore, adhesive resins must have proper fluidity allowing their infiltration into the acid-etched surface and subsequent polymerization [16].

The outcomes of this study exhibited that the viscosity results of the modified adhesive were less than that of the unmodified unfilled adhesive. Nevertheless, this difference was not statistically considerable. This finding was further reinforced by the degree of conversion results where the addition of 5 wt% BAG didn’t affect the polymerization kinetics of the adhesive and the DC results for both groups were nearly the same. This agreed with the study done by Carneiro et al., where the addition of 30 wt% niobium-based glass did not impact the degree of conversion of a commercial adhesive resin [17]. Moreover, Al-Hamdan et al. in their study showed that the addition of 5 wt% hydroxyapatite nanospheres did not affect the degree of conversion of the adhesive [26].

The BAG powder was able to release ions over the 28-day time interval (Fig. 4) and Group II adhesive showed crystal deposition. This finding agreed with Carneiro et al. where the addition of niobium-based glass (NBG) to a commercial adhesive led to the deposition of apatite precursors on the surface of the specimens [17].

Micro-tensile bond strength testing was chosen as after bonding the dentin surface will be surrounded with enamel from all sides before sectioning and the only way for the SBF to reach the dentinal surface was through the dentinal tubules and the pulp space, which is a closer simulation to the clinical condition. But in shear or micro-shear bond strength, the dentin surface surrounding the composite specimen will be exposed to the SBF.

Regarding the micro-tensile bond strength results, for both bonding techniques (ER and SE), there was no statistically significant difference between both groups after 24 h of bonding; however, after storage for 6 months, the bond strength of the unmodified adhesive decreased significantly while that of the modified adhesive remained almost unchanged.

This finding agreed with Profeta AC et al. [32], who conducted a study experimenting the effects of BAG-containing experimental adhesive on the durability of resin-dentin bonds in etch and rinse mode and concluded that there was no reduction in the micro-tensile bond strength after 6 months of storage in contrast to the control group.

This finding could be attributed to the ability of BAG to create an alkaline medium which inhibits the activation of MMPs along with creating a favorable medium for remineralization [32].

Moreover, Profeta AC et al. [32] in their testing of nanoleakage found that BAG containing experimental adhesive showed the presence of string-reflective mineral material and only partial dye penetration. This contrasted with the control group which showed severe nanoleakage and gap formation along with degradation of demineralized peritubular dentin.

The null hypothesis was rejected as the incorporation of BAG maintained the universal adhesive immediate bonding capabilities as well as the bond durability to demineralized dentin in the ER and SE modes.

A limitation of transferring the results of this study to the clinic would be the necessity of accurate weighing and proportioning of the adhesive and the BAG nanoparticles to get the desired concentration.

One of the limitations of this study was using a single concentration of BAG as well as one type of a commercially available infilled universal adhesive. Moreover, the remineralizing effect of BAG on CAD was not assessed.

Therefore, further investigation is required regarding the use of different concentrations of BAG, different commercially available adhesive systems, longer storage periods, different aging conditions as well as assessing the remineralization effect of BAG on CAD is recommended.

## Conclusions

Within the constraints of this in-vitro study, it was concluded that:

1. The addition of BAG did not alter the DC or viscosity of the adhesive.
2. Universal adhesive modified with 5 wt% BAG has maintained good immediate bonding ability to demineralized dentin.
3. BAG can enhance the bond durability of the universal adhesive to demineralized dentin in both ER & SE modes.

## Data Availability

Data will be available upon request from the corresponding author.
